# Preparation Nano-Structure Polytetrafluoroethylene (PTFE) Functional Film on the Cellulose Insulation Polymer and Its Effect on the Breakdown Voltage and Hydrophobicity Properties

**DOI:** 10.3390/ma11050851

**Published:** 2018-05-21

**Authors:** Jian Hao, Cong Liu, Yanqing Li, Ruijin Liao, Qiang Liao, Chao Tang

**Affiliations:** 1 The State Key Laboratory of Power Transmission Equipment & System Security and New Technology, Chongqing University, Chongqing 400044, China; 20171101003z@cqu.edu.cn (C.L.); cqu0926@126.com (Y.L.); rjliao@cqu.edu.cn (R.L.); 2 Postdoctoral Station of Power Engineering and Engineering Thermophysics, Chongqing University, Chongqing 400044, China; 3 College of Power Engineering, Chongqing University, Chongqing 400044, China; lqzx@cqu.edu.cn; 4 College of Engineering and Technology, Southwest University, Chongqing 400715, China; tangchao_1981@163.com

**Keywords:** cellulose insulation pressboard, magnetron sputtering, polytetrafluoroethylene, nano structure, breakdown, hydrophobicity

## Abstract

Cellulose insulation polymer is an important component of oil-paper insulation, which is widely used in power transformer. The weight of the cellulose insulation polymer materials is as high as tens of tons in the larger converter transformer. Excellent performance of oil-paper insulation is very important for ensuring the safe operation of larger converter transformer. An effective way to improve the insulation and the physicochemical property of the oil impregnated insulation pressboard/paper is currently a popular research topic. In this paper, the polytetrafluoroethylene (PTFE) functional film was coated on the cellulose insulation pressboard by radio frequency (RF) magnetron sputtering to improve its breakdown voltage and the hydrophobicity properties. X-ray photoelectron spectroscopy (XPS) results show that the nano-structure PTFE functional film was successfully fabricated on the cellulose insulation pressboard surface. The scanning electron microscopy (SEM) and X-ray diffraction (XRD) present that the nanoscale size PTFE particles were attached to the pressboard surface and it exists in the amorphous form. Atomic force microscopy (AFM) shows that the sputtered pressboard surface is still rough. The rough PTFE functional film and the reduction of the hydrophilic hydroxyl of the surface due to the shielding effect of PTFE improve the breakdown and the hydrophobicity properties of the cellulose insulation pressboard obviously. This paper provides an innovative way to improve the performance of the cellulose insulation polymer.

## 1. Introduction

Converter transformer is one of the key equipment in HVDC (High Voltage Direct Current) transmission network. The main insulation of the valve side for converter transformer usually has a high failure rate because of the complicated electric field in the operating condition, including alternating current (AC), direct current (DC), AC, and DC compound electric field [1,2]. Oil-paper insulation is the main insulation form of the valve side in converter transformer. Its insulation reliability is closely related to the safe operation of a converter transformer. The insulation performance of the insulation paper/pressboard under DC condition is an important factor to decide the operation reliability of the converter transformer.

It has been reported that polymer nanocomposites with metal oxide nanoparticle fillers can exhibit enhanced electrical breakdown strength [3,4]. In order to improve the breakdown and the mechanical properties of insulation paper, Liao Ruijin et al. developed the nano-Al_2_O_3_ doped cellulose insulation paper [5,6,7]. Results show that the nano-Al_2_O_3_ doped insulation paper possesses the better dielectric properties and AC breakdown strength. Chi Minghe et al. also studied the breakdown behavior of the Al_2_O_3_ modified pressboard [8]. Results show the breakdown strength of modified pressboard firstly increases and then decreases with the increase of nano-doping content, and reaches the peak value at 2.5% concentration. Liao Ruijin and Chi Minghe et al. also investigated the dielectric characteristics of nano-montmorillonite (MMT) modification insulation pressboard [9,10]. It is found that the breakdown strength of modified pressboard firstly increases and it then decreases with the growth of nano-doping content [5,6,7,8,9,10]. However, nanoparticle fillers research indicated that the difficulty of nano-doping is the agglomeration of nanoparticles [4,5,6,7,8,9,10]. In addition to adding nano-fillers to the material, it is worth investigating the fabrication of a special functional nano-structure film on the surface of the insulating material, which could provide an effective function for enhancing the electrical or the physicochemical properties of the oil impregnated insulation pressboard/paper that is used in the power transformer.

Moreover, moisture plays a detrimental role in the oil-paper insulation lifetime by reducing the thermal resistance and electrical breakdown strength and is regarded as “the first enemy” after temperature [11,12,13]. The production of moisture is inevitable in the service of a transformer. Field experience shows that the moisture content of a transformer is usually <0.5% in the initial stage of its operation, and it may increase to 2~4% at the last stage of its life [12]. The more moisture the insulation contains and the higher the temperature of the insulation system, the faster the oil-paper insulation material degrades [14,15]. Therefore, improving the hydrophobicity of insulating paper is very important for ensuring the performance of insulation paper.

Effective ways to improve the breakdown and hydrophobicity property of the oil impregnated insulation pressboard/paper used in power transformer are currently a popular research topic. PTFE has excellent insulation and hydrophobicity performance [16,17]. In this paper, the PTFE functional film on the cellulose insulation polymer was prepared by RF magnetron sputtering. Firstly, the structure and the existence form of the PTFE functional film were characterized. Then, the effect of the PTFE functional film on the breakdown and the hydrophobicity properties of the cellulose polymer were analyzed.

## 2. Materials and Methods

### 2.1. Materials and Sample Preparation

**Figure 1:**
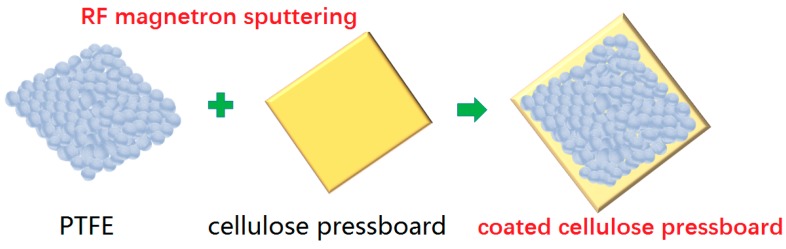
RF magnetron sputtering polytetrafluoroethylene (PTFE) functional film on the cellulose pressboard surface.

The cellulose insulation pressboard with a thickness 0.45 mm was used at here. The JPGF-480 reactive RF magnetron sputtering device at 13.56 MHz (Beijing Instrument Factory, Beijing, China) was used for pressboard coating. For PTFE film deposition, the insulation pressboard substrates were cut into 15 cm × 10 cm pieces, and a PTFE target (diameter 61.5 mm, thickness 5 mm) was sputtered. The distance between the target and the substrate sample was 10 cm. The vacuum chamber was pumped down to a base pressure of 4.0 × 10^−3^ Pa before sputtering. Deposition was conducted by using forward power 100 W. Argon was used as the working gas with a constant pressure of 1.5 Pa. The deposition mode was static, double-sided coating, and the deposition time was 10 min and 20 min at 28 °C. The sketch map for the RF magnetron sputtering PTFE functional film on the cellulose pressboard surface is shown in Figure 1. The sample composition is shown in Table 1.

### 2.2. Characterization Methods and Sample Treatment

X-ray photoelectron spectroscopy (XPS) with Al Kα X-ray source (XPS, Thermo escalab 250Xi, Waltham, MA, USA) was used to characterize the chemical binding state of the deposition film. The XPS spectra without argon etching were recorded in the fixed analyzer transmission mode with pass energy of 20 eV and a resolution of 0.1 eV. The deviation that is caused by the charging effect was calibrated using adventitious carbon referencing (C 1s, 284.6 eV). The scanning electron microscopy (SEM) (JSM-7800F, JEOL, Tokyo, Japan) and atomic force microscopy (AFM, Bruker Daltonics Inc., Billerica, MA, USA) were used to investigate the surface morphology of the coated surface. The X-ray diffractometer using Cu Ka (λ = 0.154 nm) radiation at a fixed incident angle of 2° was used to obtain the X-ray diffraction (XRD) (PANalytical Empyrea, Almelo, The Netherlands) patterns of the samples.

**Figure 2:**
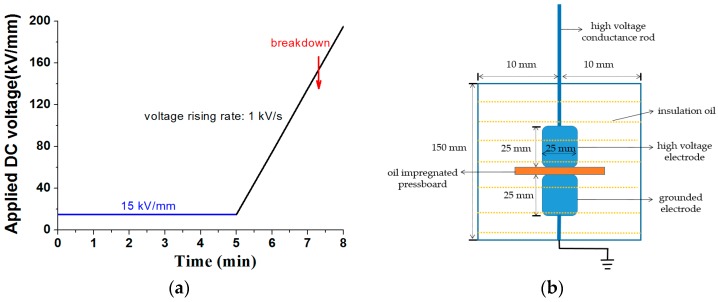
Direct current (DC) breakdown test process and the DC breakdown test electrode setup. (**a**) DC breakdown test process; (**b**) DC breakdown test electrode setup.

Before the DC (direct current) breakdown experiment, firstly, all of the samples were dried at 90 °C for 24 h in a vacuum box (1000 Pa). Then, new mineral oil was infused into the vacuum box and the temperature of the vacuum box was adjusted to 40 °C. The pressboard was impregnated at 40 °C for 48 h. The parameter of the oil used for impregnation is shown in Table 2. The measured moisture content of the oil-impregnated pressboard using Karl Fischer titration method was 0.95% after impregnation, and being cooled to room temperature. The DC breakdown voltage was measured according to Figure 2a. The pre-pressure DC voltage (15 kV/mm) was applied for 5 min. Then, the voltage was increased at 1 kV/s until sample breakdown. Five breakdown voltages were recorded for each sample. The breakdown test electrode setup is shown in Figure 2b. The test temperature is 28 °C.

At last, the contact angle was measured with a Kyowa contact angle meter. Three measurements on different sample spots were made for each specimen. An average of the measurements was used for analysis. For XPS, SEM, AFM, XRD and contact angle test, non-impregnated pressboard samples were used. For the DC breakdown voltage test, the oil impregnated insulation pressboard was used.

## 3. Results and Discussions

### 3.1. XPS Analysis

**Figure 3:**
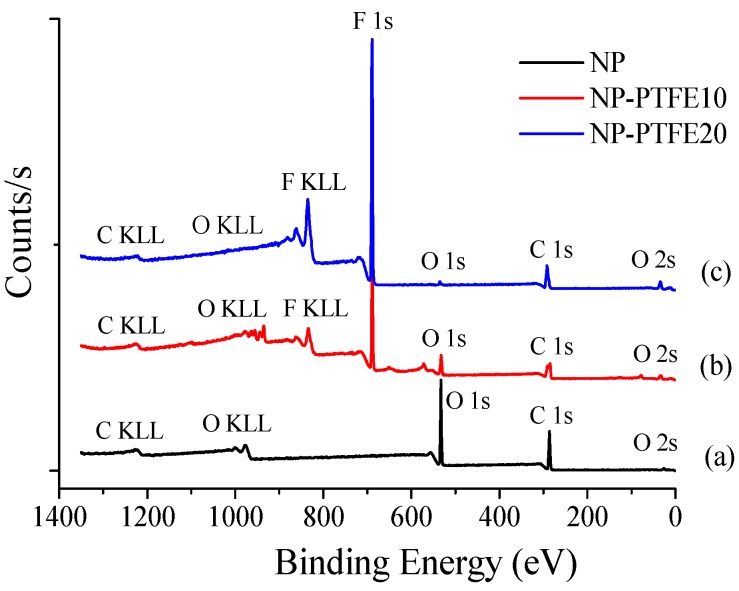
The X-ray photoelectron spectroscopy (XPS) spectra of the new pressboard (**a**), new pressboard surface as-prepared. PTFE film for 10 min (**b**) and 20 min (**c**).

Figure 3 shows the XPS survey spectra of the new pressboard, new pressboard surface as-prepared PTFE film for 10 min and 20 min. Cellulose insulation pressboard consists of linear, polymeric chains of cyclic, β-d-glucopyranose units, which are composed of C, H, and O element [18]. Therefore, there is only C 1s and O 1s peak, and extremely weak O 2s peak shown in Figure 3a. The molecular formula of PTFE is (C_2_F_4_)n. As shown in Figure 3b,c, it is obvious that the F 1s peak appears on the pressboard surface as-prepared PTFE film for 10 min and 20 min. With the increase of the coating time, the F 1s peak is obviously enhanced [19]. While the O 1s peak becomes weaker and weaker because of the coverage of the PTFE film on the surface of the cellulose pressboard. The O 1s peak almost disappeared for the pressboard surface deposited PTFE 20 min.

**Figure 4:**
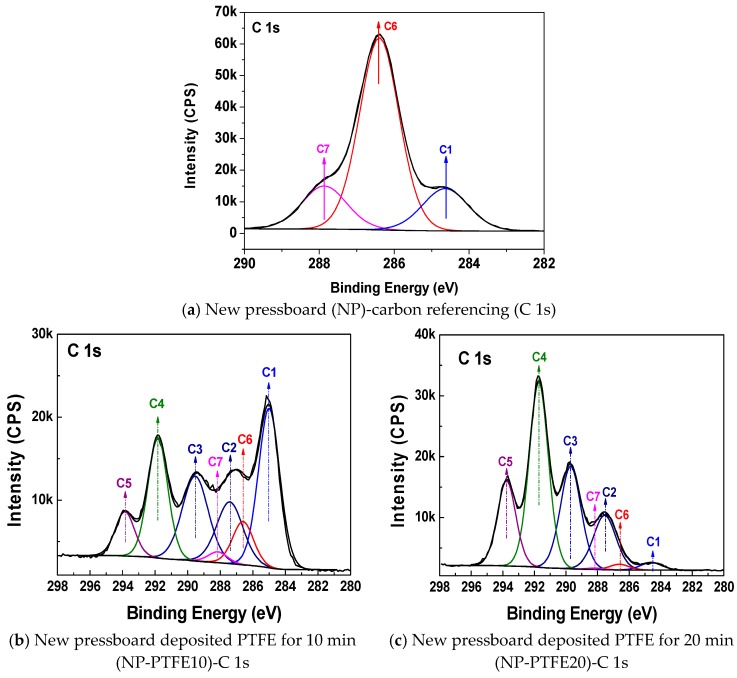
C 1s peak fitting for the new pressboard (**a**), new pressboard coated PTFE film for 10 min (**b**), and 20 min (**c**).

**Figure 5:**
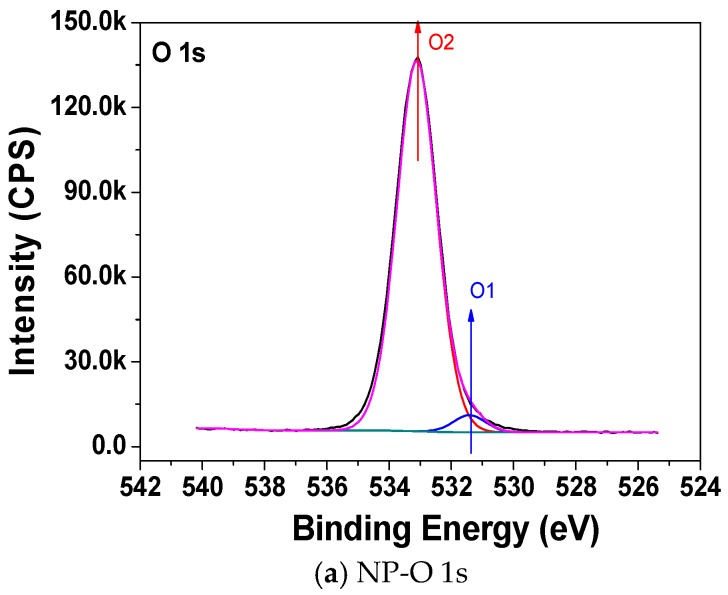

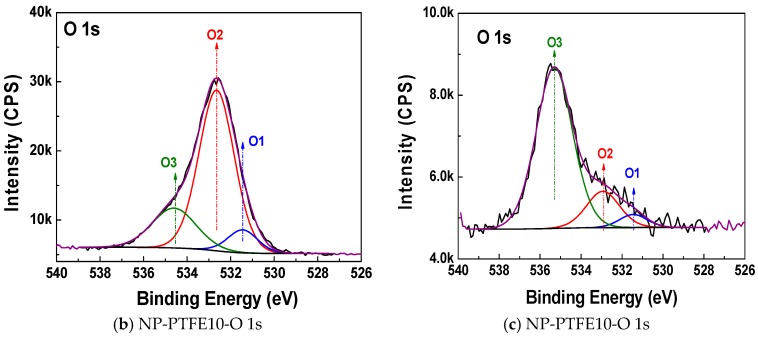
O 1s peak of the new pressboard, pressboard surface coated PTFE film for 10 min and 20 min.

**Figure 6:**
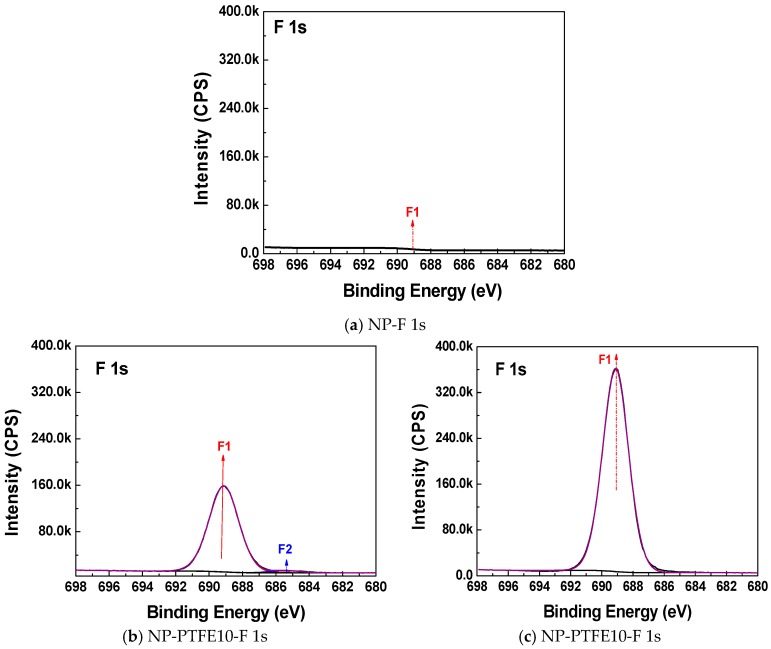
F 1s peak of the new pressboard, pressboard surface coated PTFE film for 10 min and 20 min.

Figure 4, Figure 5 and Figure 6 show the C 1s, O 1s, and F 1s peak fitting in the XPS narrow scan spectra, respectively. The peak fitting can be used to make identify the chemical components. The identified chemical components with different binding energies and its concentration by C 1s, O 1s, and F 1s peak fitting are shown in Table 3, Table 4 and Table 5, respectively. The C1, C6, and C7 peaks that are presented in Figure 4a are attributed to C–C/C=C, C–O, and O–C=O, respectively [19,20]. It is particularly noteworthy from Figure 4b,c that new C2, C3, C4, C5 peaks appear for the sample NP-PTFE10 and NP-PTFE20. The C2, C3, C4, C5 peaks are attributed to O–C–CF_3_/CF_2_, CF, CF_2_, and CF_3_, respectively [19,20]. From the O 1s high resolution spectra shown in Figure 5b,c, it can be seen that new O3 peak appears for the coated samples. The O1 and O2 are attributed to O=C–O and O–C. The O3 is attributed to O–C–CF_3_/CF_2_ [19,20]. Figure 6b,c show that there are also new F1 peak attributed to F–C appears for the pressboard surface deposited PTFE for 10 min and 20 min. With the coating time increase, due to the covering PTFE film, Figure 4b,c show that the intensity of the new C3, C4, C5 peaks becomes stronger, while the intensity of C1, C6, and C7 peaks becomes weaker. The intensity of new F 1 peaks for the pressboard surface as-prepared PTFE film for 20 min is significantly stronger than that of the sample as-prepared PTFE film for 10 min. From Figure 5 and Figure 6, Table 3, Table 4 and Table 5, it could be deduced that the PTFE has been successfully fabricated on the cellulose insulation pressboard surface.

### 3.2. Surface Topography Analysis

**Figure 7:**
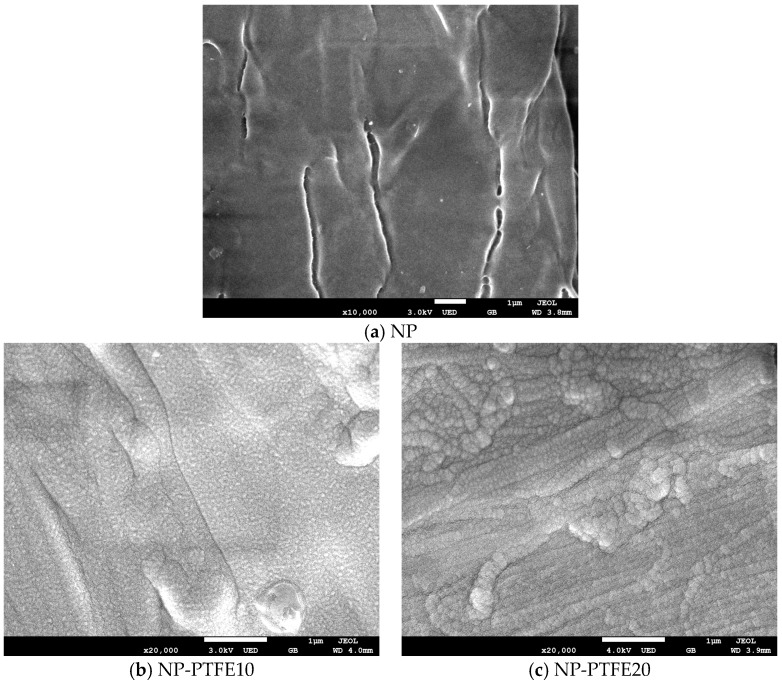
The scanning electron microscopy (SEM) of the new pressboard, pressboard surface coated PTFE film for 10 min and 20 min.

The SEM micrographs of the untreated pressboard and the coated pressboard are shown in Figure 7. We can observe that the cellulose fibers of untreated pressboard (Figure 7a) intersect each other and its surface is relatively rough. There are some cracks where the fibers intersect. The pressboard surface with magnetron sputtering treatment for 10 min (Figure 7b) is more smooth and dense. There are many very small PTFE particles with nanometer covered on the surface. The PTFE particles filled the cracks between the fibers and were distributed uniformly on the surface. However, for the pressboard surface sputtered for 20 min, as shown in Figure 6c, the cracks also are be filled. Besides, PTFE is present in larger particles due to the agglomeration of particles. The PTFE particles are about a few dozen nanometers in size.

**Figure 8:**
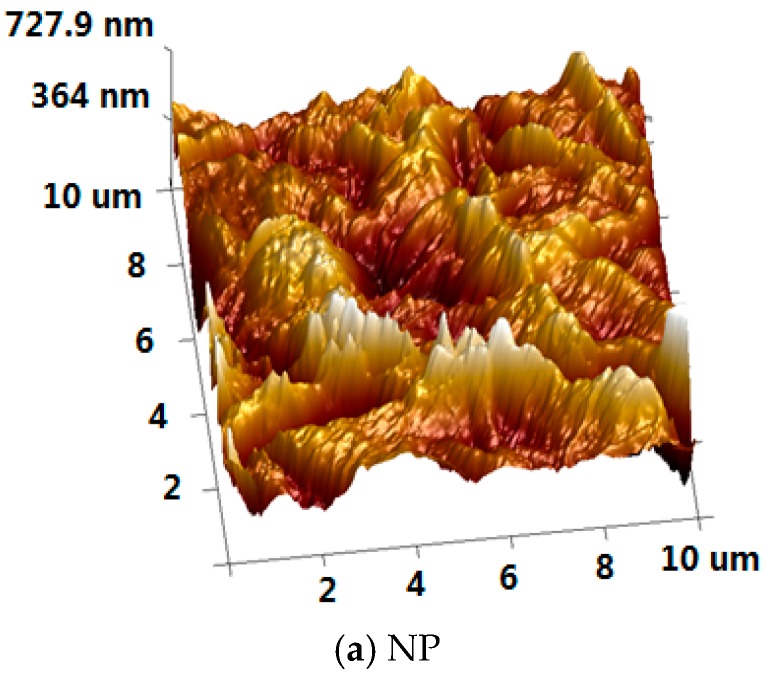

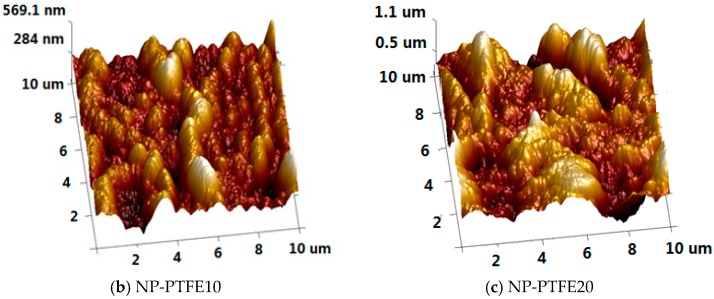
The atomic force microscopy (AFM) of the new pressboard, pressboard surface coated PTFE film for 10 min and 20 min.

The microscopic appearance of insulating pressboard specimen before and after magnetron sputtering was measured by AFM (Figure 8). The AFM image shows a greater longitudinal undulating and some sharp protrusions, in good agreement with the SEM micrograph revealing a relatively rough surface. After specimen treatment, there are some obvious changes that have taken place. By comparing the fresh pressboard, we can find that the pressboard surface coated PTFE for 10 min (Figure 8b) is smoother than that of new pressboard, as well as the raised part is granular and relatively flat. Figure 8c shows the surface topography of sample that is modified by magnetron sputtering for 20 min, and as the sputtering time increases, the sample surface becomes slightly rougher again.

### 3.3. XRD Analysis

**Figure 9:**
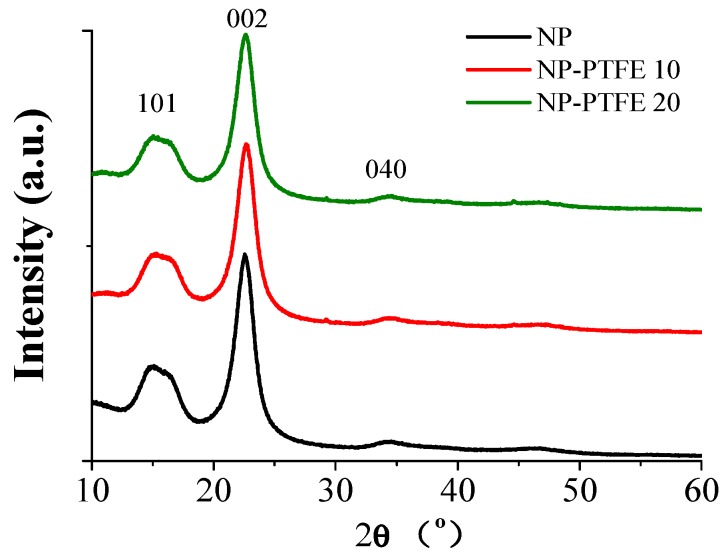
X-ray diffraction (XRD) of the the new pressboard, pressboard surface coated PTFE film for 10 min and 20 min.

Figure 9 shows the XRD spectrum of new pressboard and the PTFE coated pressboard. Diffraction pattern for the pressboard has three broad peaks at 2θ = 15°, 2θ = 22°, and 2θ = 34°, corresponding to (101), (002), and (040) diffraction peaks of cellulose, respectively [21]. In the diffraction pattern of the new pressboard, there is a sharp peak and some dispersive diffraction peaks, which means that the cellulose has a mixed structure of crystallization and amorphous phase. The diffraction peak of PTFE is at 17°, 30°, and 35° [22]. We can notice that there is no peak at 2θ = 17°, 2θ = 30°, and 2θ = 35° in the XRD results of coated pressboard, which proves that the PTFE film exists on the surface of insulation pressboard surface in the amorphous form.

### 3.4. DC Breakdown Analysis

**Figure 10:**
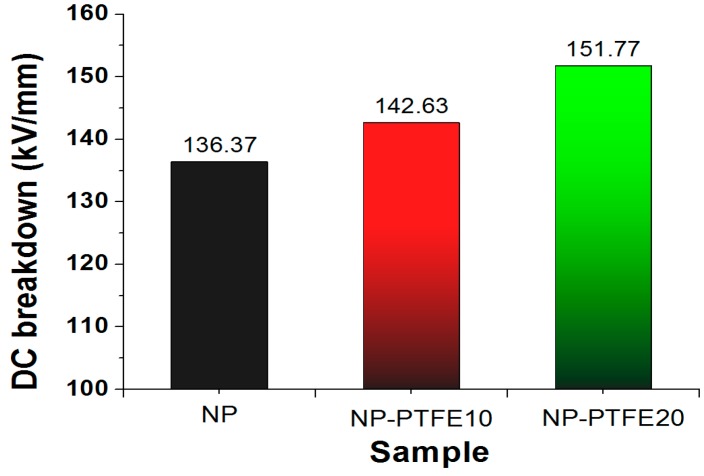
DC pre-pressure breakdown strength of the new pressboard, new pressboard deposited. PTFE for 10 min and 20 min.

The DC pre-pressure breakdown strength of the new pressboard (NP), new pressboard deposited PTFE for 10 min and 20 min (NP-PTFE10, NP-PTFE20) is shown in Figure 10. The “pre-pressure breakdown strength” means the breakdown voltage obtained through the test process shown in the Figure 2. The average DC pre-pressure breakdown voltage for the NP, NP-PTFE10 and NP-PTFE20 is 136.37 kV/mm, 142.53 kV/mm, and 151.77 kV/mm, respectively. When compared with the new pressboard, the DC pre-pressure breakdown enhancement is 5% and 11% for NP-PTFE10 and NP-PTFE20, respectively. The PTFE functional film improves the breakdown property of the coated insulation pressboard, especially for the sample NP-PTFE20. This mainly because the nano PTFE particles filled the surface defects (Figure 7) and improved the breakdown performance.

### 3.5. Hydrophobicity and Hygroscopicity Analysis

**Figure 11:**
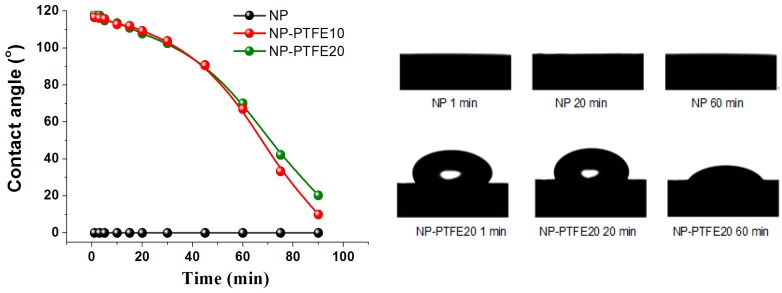
The contact angle of the new pressboard, new pressboard deposited PTFE for 10 min and 20 min.

The insulation paper was developed from natural fiber. The structural characteristics of the fiber determines that it absorbs water very easily. However, the hygroscopicity of the insulation paper is a very bad feature when it is being used for insulation in the transformer. Therefore, if the insulation paper has better hydrophobicity, its performance is not easily destroyed by moisture. Figure 11 shows the detailed dynamic process of liquid droplets that are dripping on the surface of each sample. The contact angle is about 0° that it is impossible to measure, indicating that water droplet penetrated through the surface of pressboard due to the hydrophilicity of cellulose. However, the pressboard surface coated by PTFE shows hydrophobicity. Water droplets can last a long time on the PTFE surface. As reported in [23,24,25], the surface hydrophobicity should increase in the order –CH_2_ < –CH_3_ < –CF_2_ < –CF_2_H < –CF_3_. The pressboard surface coated PTFE has much C–F groups which improve its hydrophobicity. At the beginning, the contact angle of pressboard deposited PTFE for 10 min and 20 min is 118.2° and 116.6°, respectively. As time increases, the contact angle decreases gradually. Before 45 min, both of the samples have the same change, and both are greater than 90°. Then, the sample contact angle of pressboard surface as-prepared PTFE film for 10 min decreased faster than the PTFE film coated for 20 min.

**Figure 12:**
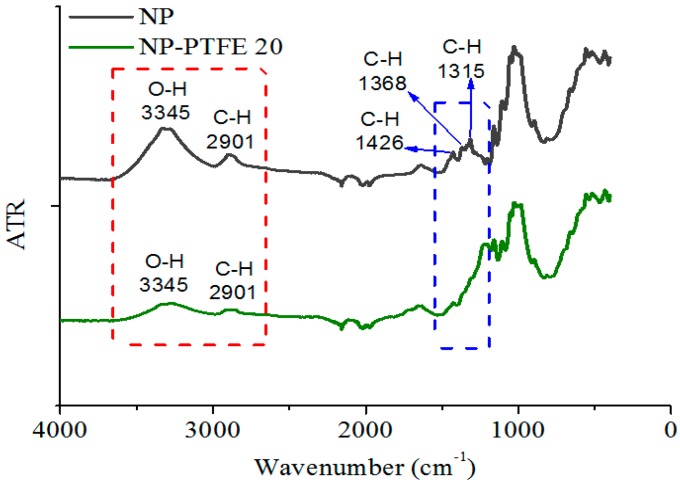
FT-IR of the new pressboard, new pressboard deposited PTFE for 20 min.

PTFE is a non-polar polymer with symmetrical structure, and it is one of the lowest surface energy materials [19,20]. In order to explain the change of surface hydrophobicity from the chemical mechanism, the Fourier transform infrared spectroscopy (FT-IR) spectroscopy (Nicolet iS5 FT-IR) was used to confirm the reason for the change of the contact angle. FT-IR analysis was further carried out. As shown in Figure 12, the peak at 3345 cm^−1^ is assigned to the stretching vibration of O–H [26,27]. The peak at 2901 cm^−1^, 1426 cm^−1^, 1368 cm^−1^, and 1315 cm^−1^ is assigned to the stretching vibration and the flexural vibration of C–H [26,27]. It is obvious that the shielding of nano-structure PTFE film leads to the reduction of hydroxyl, which is beneficial to reduce the interaction between hydroxyl and water. In addition, PTFE has the excellent hydrophobic and oleophylic properties [19,20]. The above two aspects increase the contact angle of the sputtered insulation pressboard surface.

**Figure 13:**
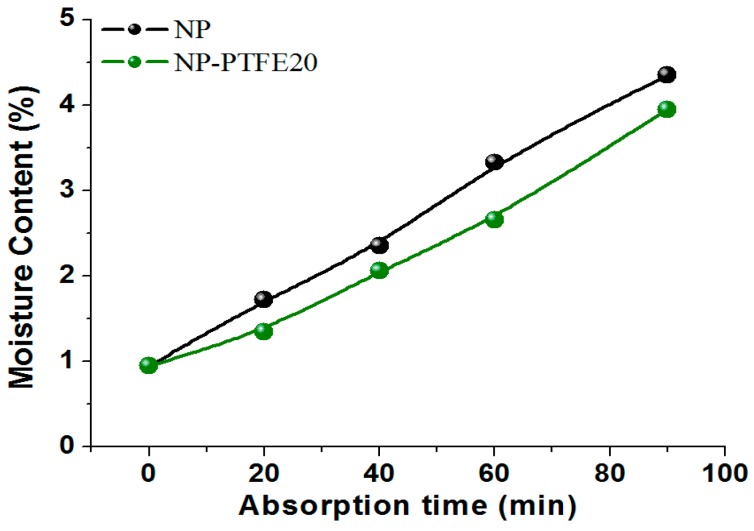
Moisture content of the NP and NP-PTFE20 samples under the absorption moisture condition.

The hygroscopicity of the dried new pressboard and the pressboard coated for PTFE was also compared at here. According to the moisture equilibrium experiment that was done by our team [28], the dried pressboard samples were placed into a humidity chamber. The temperature of the humidity chamber was set to 60 °C and the relative humidity was set to 60%. The absorption time was set as 0 min, 20 min, 40 min, 60 min, and 90 min. The moisture content of the dried new pressboard (NP) and the pressboard coated for PTFE (NP-PTFE20) is shown in Figure 13. It can be seen from Figure 13 that the moisture absorption rate for NP-PTFE20 sample is slower than that of the NP sample.

**Figure 14:**
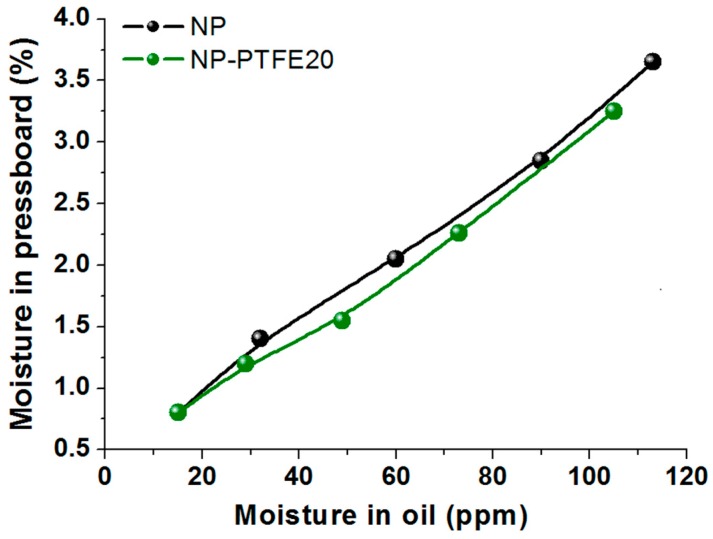
Moisture balance between paper and oil at 70 °C.

For the moisture balance between paper and oil, the pressboard samples absorbed moisture for different times (0 min, 20 min, 40 min, 60 min, and 90 min) were placed into grinding bottles that were filled with new insulating oil. The grinding bottles were then sealed and placed in a constant temperature oven under controlled temperature 70 °C. The moisture concentration in oil and paper was constantly measured until the equilibrium state was considered to be achieved, when the measured moisture concentration remained constant. In this paper, the time that is required for reaching moisture equilibrium state was 13 days at 70 °C. The result for the moisture balance between paper and oil is shown in Figure 14. It can be seen that for the NP-PTFE20 sample, the moisture tends to stay in the oil.

### 3.6. Oil Absorption and Impregnation

**Figure 15:**
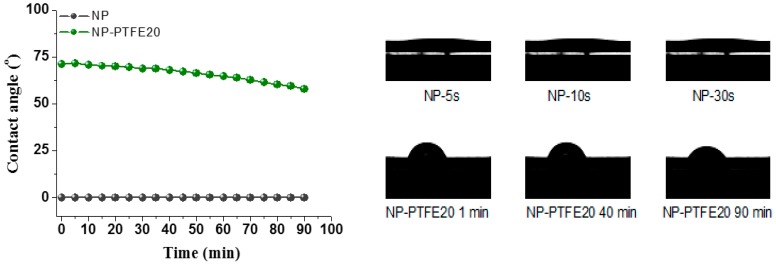
Contact angle between oil and paperboard.

In order to investigate the PTFE functional film influence on the oil impregnation, we measured the contact angle between oil and paperboard, and compared the process of oil drop that is entering into the pressboard with and without PTFE functional film. As shown in Figure 15, the NP-PTFE20 sample has higher contact angle between oil and paperboard. The oil drop entering into the NP is very quickly, while the oil drop entering into the NP-PTFE20 sample is very slow. It takes about 3 h to fully enter the interior of the NP-PTFE20 sample. Therefore, the oil impregnation process is slow for the pressboard PTFE functional film.

**Figure 16:**
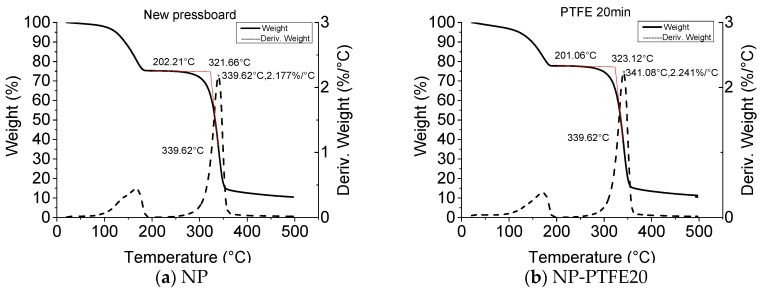
TG for NP and NP-PTFE20.

In order to obtain the difference in the amount of oil that is impregnated into the NP and NP-PTFE 20 sample, the thermogravimetry (TG) and the derivative thermogravimetry (DTG) curves of the NP and NP-PTFE 20 sample impregnated with oil was measured, as shown in Figure 16. The heating rate is 7 °C/min. Each sample is 5.0 mg. The tested temperature is from 33 °C to 500 °C under a nitrogen flow of 50 mL/min. There are two peaks that can be seen for both NP and NP-PTFE20. This is because there is oil in the oil impregnated pressboard, and the thermal properties of oil and the pressboard are different. The first stage of weight loss in TG curve and the first peak in DTG curve is belonging to oil decomposition [29]. Subtracting the 0.95% moisture content, it can be deduced in Figure 16 that the NP sample contain 23.86% oil in the oil impregnated pressboard (mass ratio). The NP-PTFE20 sample contain 21.28% oil in the oil impregnated pressboard (mass ratio). The PTFE film fills many gaps on the surface, resulting in oil absorption being reduced.

**Figure 17:**
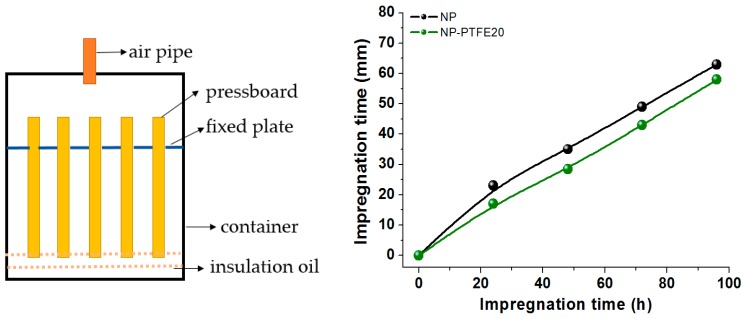
Oil impregnation experiment and result.

For the oil impregnation experiment, the impregnation test model is shown in Figure 17. The size of the pressboard is 100 mm × 30 mm × 0.5 mm. The impregnation experiment was conducted at 30 °C, 1 atm. The impregnation length versus the impregnation time is shown in Figure 17. The samples that were used was new pressboard (NP) and new pressboard coated PTFE for 20 min (NP-PTFE20). Because the PTFE was coated on the two side surface of the pressboard, the oil impregnated the inner part from the bottom and other side surface of the samples where there is no PTFE. Thus, the oil impregnation rate of NP-PTFE20 is slower than that of NP sample.

## 4. Conclusions

The present study confirmed the finding about improving the DC pre-pressure breakdown and the hydrophobicity properties of the cellulose insulation polymer by sputtering nano-structure PTFE functional film on the surface. The conclusions are as follows:

The nano-structure PTFE functional film was successfully fabricated on the cellulose insulation pressboard surface by RF magnetron sputtering. When compared with the fresh cellulose insulation pressboard, for the pressboard sputtered PTFE for 10 min and 20 min, the new peaks attributed to O–C–CF_3_/CF_2_, CF, CF_2_, and CF_3_ appear in their C 1s XPS spectroscopy, the new peak that is attributed to O–C–CF_3_/CF_2_ appears in the O 1s XPS spectroscopy, and for F 1s XPS spectroscopy, the new peaks that are attributed to F–C, F_2_–C appear.

The SEM and XRD present that the nanoscale size PTFE particles were attached on the pressboard surface and exists in the amorphous form. The PTFE particles are about a few dozen nanometers in size for the surface sputtering 20 min. There are only three broad XRD peaks at 2θ = 15°, 2θ = 22° and 2θ = 34°, which belongs to cellulose. AFM result shows that the sputtered pressboard surface is still rough.

The DC pre-pressure breakdown enhancement is 5% and 11% for NP-PTFE10 and NP-PTFE20, respectively. The contact angle of the new pressboard is 0°. However, the cellulose pressboard surface deposited PTFE for 10 min and 20 min is 118.2°and 116.6°, respectively. FTIR spectroscopy shows that the transmissivity of the peak at 3345 cm^−1^ for O–H and the peaks at 2901 cm^−1^, 1426 cm^−1^, 1368 cm^−1^, and 1315 cm^−1^ for C–H decreases for the sputtered sample. The rough PTFE functional film and the reduction of the hydrophilic hydroxyl of the surface due to the shielding effect of PTFE improve the DC pre-pressure breakdown and hydrophobicity properties of the cellulose insulation pressboard obviously.

The moisture absorption rate for NP-PTFE20 sample is slower than that of the NP sample. According to the result of the moisture balance between paper and oil, it shows that the moisture tends to stay in the oil. The NP sample contains 23.86% oil in the oil impregnated pressboard (mass ratio), and the NP-PTFE20 sample contains 21.28%. The oil impregnation rate of NP-PTFE20 is slower than that of the NP sample.

## Figures and Tables

**Table 1:** Sample composition.

Sample	Abbreviation
new pressboard	NP
new pressboard deposited PTFE for 10 min	NP-PTFE10
new pressboard deposited PTFE for 20 min	NP-PTFE20

**Table 2:** The parameter of the oil used for impregnation.

Property	Mineral Oil
Kinematic viscosity 40 °C (mm^2^/s)	9.7
Acidity (mg KOH/g)	0.008
Breakdown voltage (2.5 mm gap electrodes) (kV)	47.0
Relative permittivity (50 Hz)	2.2
Moisture content (ppm)	9

**Table 3:** C 1s peak fitting result for NP, NP-PTFE10, and NP-PTFE20.

Sample	Data Set	Name	Position	% Conc.
NP	C–C/C=C	C1	284.6	16.9
	C–O	C6	286.4	66.2
	O–C=O	C7	287.9	16.9
NP-PTFE10	C–C/C=C	C1	285.0	29.4
	O–C–CF_3_/CF_2_	C2	287.4	14.0
	C–F	C3	289.5	19.2
	C–F_2_	C4	291.8	20.5
	C–F_3_	C5	293.9	7.3
	C–O	C6	286.6	7.7
	O–C=O	C7	288.2	1.8
NP-PTFE20	C–C/C=C	C1	284.6	1.9
	O–C–CF_3_/CF_2_	C2	287.6	14.0
	C–F	C3	289.7	24.2
	C–F_2_	C4	291.7	40.3
	C–F_3_	C5	293.8	18.0
	C–O	C6	286.6	1.2
	O–C=O	C7	288.2	0.3

**Table 4:** O 1s peak fitting result for NP, NP-PTFE10, and NP-PTFE20.

Sample	Data Set	Name	Position	% Conc.
NP	O=C–O	O1	531.4	3.6
	O–C	O2	533.1	96.4
NP-PTFE10	O=C	O1	531.4	8.4
	O–C	O2	532.6	69.9
	O–C–CF_3_/CF_2_	O3	534.6	21.7
NP-PTFE20	O=C	O1	531.4	4.8
	O–C	O2	532.9	16.2
	O–C–CF_3_/CF_2_	O3	535.3	79.0

**Table 5:** F 1s peak fitting result for NP, NP-PTFE10, and NP-PTFE20.

Sample	Data Set	Name	Position	% Conc.
NP	/	/	/	/
NP-PTFE10	F_2_–C	F1	689.1	97.5
	F–C	F2	685.4	2.5
NP-PTFE20	F_2_–C	F1	689.1	100.0
